# Safety and efficacy of first-line iparomlimab and tuvonralimab (QL1706) plus carboplatin and etoposide in patients with extensive-stage small cell lung cancer: an open-label, single-arm, phase 2 trial (DUBHE-L-209)

**DOI:** 10.3389/fimmu.2026.1879524

**Published:** 2026-08-11

**Authors:** Yun Fan, Runxiang Yang, Xuhong Min, Huiwen Ma, Yan Wang, Yanqiu Zhao, Hongmin Wang, Mingjun Zhang, Huayuan Wang, Tao Zhang, Zhehai Wang

**Affiliations:** 1 Department of Thoracic Medical Oncology, Zhejiang Cancer Hospital, Hangzhou, China; 2 Department of Internal Medicine II, Yunnan Cancer Hospital, Kunming, China; 3 Department of Tumor Radiotherapy, Anhui Chest Hospital, Hefei, China; 4 Department of Medical Oncology, Chongqing University Cancer Hospital, Chongqing, China; 5 Department of Respiratory Medicine, Harbin Medical University Cancer Hospital, Harbin, China; 6 Department of Respiratory Medicine, Henan Cancer Hospital, The Affiliated Cancer Hospital of Zhengzhou University, Zhengzhou, China; 7 Department of Respiratory Medicine, The First Affiliated Hospital of Zhengzhou University, Zhengzhou, China; 8 Department of Medical Oncology, The Second Affiliated Hospital of Anhui Medical University, Hefei, China; 9 Qilu Pharmaceutical Co., Ltd., Jinan, China; 10 Department of Respiratory Medicine, Shandong Cancer Hospital, Jinan, China

**Keywords:** chemotherapy, DUBHE-L-209, immunotherapy, QL1706, small cell lung cancer

## Abstract

**Background:**

Iparomlimab and tuvonralimab (QL1706) is a MabPair product consisting of PD-1 and CTLA-4 monoclonal antibodies. This single-arm, multicenter, phase 2 study assessed the safety and efficacy of first-line QL1706 plus etoposide and carboplatin (EC) for extensive-stage small cell lung cancer (ES-SCLC).

**Methods:**

Patients with ES-SCLC received QL1706 plus EC every 3 weeks for 4–6 cycles, followed by QL1706 maintenance therapy until disease progression. Primary endpoint was safety.

**Results:**

Among 40 patients enrolled, all patients experienced at least one treatment-related adverse event (TRAE). Most TRAEs were hematologic toxicities. Nine patients (22.5%) experienced immune-related adverse events, mostly grade 1–2, with a median time from treatment to the first irAE of 3.1 months (range 0.1–11.0) with a median duration of the irAEs of 1.3 months (range 0.1–10.7). Grade 3 irAEs occurred in 2 (5.0%) patients, one case each for rash and vomiting. No TRAEs leading to death or treatment discontinuation occurred. In 38 patients evaluable for efficacy, the confirmed objective response rate was 92.1% (95% confidence interval [CI] 78.6–98.3). Median progression-free survival (PFS) and overall survival (OS) were 6.0 months (95% CI 5.4–8.3) and 15.8 months (95% CI 11.4–20.0), respectively. In exploratory analyses, the median OS was 20.5 months (95% CI 11.4–not evaluable) in patients with a good lung immune prognostic index status and 19.7 months (95% CI 11.4–22.5) in patients with a combined positive score <1.

**Conclusions:**

QL1706 plus EC was well-tolerated and showed promising anti-tumor activity and survival benefit in first-line treatment for ES-SCLC, and warranted further validation in larger scale phase 3 studies.

## 1 Introduction

Small cell lung cancer (SCLC) is a high-grade neuroendocrine carcinoma with an exceptionally poor prognosis, which represents 10% to 15% of all lung cancers and is strongly associated with tobacco smoking, as approximately 95% of patients with SCLC have a history of tobacco use (1–3). About 60% of patients with SCLC may be asymptomatic at initial diagnosis, and approximately 70% of patients with SCLC were initially diagnosed with extensive-stage SCLC (ES-SCLC) according to the Veterans Administration Lung Study Group (VALG) staging system, with no chance of curative multimodality therapy (1).

SCLC cells exhibit high tumor mutation burden (TMB), which is predictive of robust T-cell responses and enhanced responsiveness to immunotherapy (1). Currently, anti-programmed cell death ligand 1 (PD-L1)/programmed cell death protein 1 (PD-1) antibodies plus standard chemotherapy, followed by maintenance immunotherapy have brought tangible progress in the frontline management of ES-SCLC with an overall survival (OS) benefit of 2.0–4.5 months (4–11). To date, chemoimmunotherapy with anti-PD-L1 antibodies, i.e. atezolizumab and durvalumab, has gained global approval, while chemoimmunotherapy with other PD-1/PD-L1 antibodies, including adebrelimab, serplulimab, tislelizumab, and toripalimab, has only been approved in China (10). Beyond the geographic disparities in the approval of immune checkpoint inhibitors (ICIs) for first-line ES-SCLC treatment, the current standard chemoimmunotherapy with PD-1/PD-L1 antibodies confers a median OS of 10.8–15.5 months and a response rate of 60.2%–80.2% in first-line treatment of ES-SCLC patients (4–9, 11), and the 3-year and 5-year OS rates were 17.6% and 12.0%, respectively (12–14). Thus, there is still an urgent need for novel first-line chemoimmunotherapy regimens to improve outcomes in patients with ES-SCLC (15).

Dual PD-1/PD-L1 and cytotoxic T lymphocyte–associated protein 4 (CTLA-4) blockade plus chemotherapy remains a rational chemoimmunotherapy strategy for SCLC. Dual inhibition of PD-1/PD-L1 and CTLA-4 synergistically activates naive/resting T cells during the priming stage to restore the activity of effector T cells in the tumor microenvironment (TME), leading to enhanced anti-tumor T-cell responses (16, 17). Additionally, CTLA-4 inhibition may increase the compensatory expression of PD-L1 on tumor cells and mediate a switch from expansion of exhausted CD8^+^ T cells to expansion of activated effector CD8^+^ T cells (18). However, in the CASPIAN trial, the addition of durvalumab and tremelimumab (an anti-CTLA-4 antibody) to standard etoposide-platinum (EP) chemotherapy in first-line treatment for ES-SCLC was not associated with significant improvement in OS (14). The considerably increased treatment-related toxicities in the durvalumab + tremelimumab + EP arm partly resulted in lower exposure to all three therapeutic components (chemotherapy, durvalumab, and tremelimumab), which contributed to the reduced objective response rate (ORR; 68% [durvalumab + EP] *vs* 58% [durvalumab + tremelimumab + EP] *vs* 58% [EP only]), and ultimately diminished median OS benefit compared with EP alone (OS: 12.9 months [durvalumab + EP] *vs* 10.4 months [durvalumab + tremelimumab + EP] *vs* 10.5 months [EP only]) (5, 12, 14). Importantly, the therapeutic limitations of this combination regimen are further compounded by a lack of reliable predictive biomarkers, which prevents optimal patient selection for this intensive immunotherapy approach. To characterize heterogeneous responses to first-line chemoimmunotherapy in ES-SCLC, multiple biomarker approaches have been investigated, including baseline lung immune prognostic index (LIPI) (13, 19–22). Despite these multidimensional exploratory efforts encompassing molecular, immunological and clinical parameters, no biomarker has yet been validated to reliably predict treatment outcomes of chemoimmunotherapy in ES-SCLC.

The unmet clinical need in ES-SCLC has spurred investigation of first-line dual-ICI-chemoimmunotherapy. The combination of iparomlimab and tuvonralimab (QL1706) is a bifunctional antibody mixture consisting of anti-PD-1 and anti-CTLA-4 components and has shown promising anti-tumor activity in multiple solid tumors (23). The phase 1 study showed that QL1706 monotherapy had a favorable overall and long-term safety profile, characterized by a low incidence of grade 3–4 treatment-related adverse events (TRAEs) of 18.9% in all patients and 24.7% in those receiving treatment for ≥12 months (24). Additionally, QL1706 combined with chemotherapy has been approved for patients with platinum-resistant recurrent/metastatic cervical cancer by the National Medical Products Administration in China. Based on the favorable safety profile and promising anti-tumor activity of QL1706 in ES-SCLC patients in the phase 1 study (23, 25), we designed this phase 2 study (DUBHE-L-209) to evaluate the safety and efficacy of QL1706 combined with etoposide and carboplatin (EC) as first-line treatment for ES-SCLC.

## 2 Methods

### 2.1 Study profile

This was an open-label, multicenter, single-arm, phase 2 study conducted at nine sites in China. The study was conducted in accordance with the principles of Good Clinical Practice and the Declaration of Helsinki, and the International Council for Harmonization of Technical Requirements for Pharmaceuticals for Human Use guidelines. Approvals for the trial protocol (and any amendments) were obtained from independent ethics committees of each participating center, as well as from the Institutional Review Board of Zhejiang Cancer Hospital ethics (approval No. IRB-[2022]372) and the Institutional Review Board of Shandong Cancer Hospital (ethics approval No. SDZLEC2022-080-01). All patients provided written informed consent prior to study procedures. This study was registered on ClinicalTrials.gov (NCT05309629).

### 2.2 Patients

This study enrolled patients aged 18 to 75 years with histologically or cytologically confirmedES-SCLC, staged according to the VALG staging system. Key inclusion criteria were Eastern Cooperative Oncology Group (ECOG) performance status 0 or 1; measurable lesions per Response Evaluation Criteria in Solid Tumors (RECIST) version 1.1; a life expectancy of at least 12 weeks; and adequate organ and bone marrow function. All included patients provided biopsy samples or archived tumor samples that were acquired before the first dose of study treatment. Patients were excluded if they had active or untreated central nervous system metastases, active autoimmune diseases, received corticosteroids (> 10 mg/day prednisone or equivalent dose) or other immunosuppressants within 14 days before first dose of study treatment; had a history of idiopathic pulmonary fibrosis or active inflammatory lung diseases; had evidence of active infections that required systemic therapy within 7 days prior to the first dose of study treatment; or received live-attenuated vaccines within 28 days before screening (patients who received an inactivated COVID-19 vaccine were allowed to enroll). Full inclusion and exclusion criteria are detailed in **Supplementary Table 1**.

### 2.3 Study treatment

Patients received 4–6 cycles of chemoimmunotherapy consisting of QL1706 (5 mg/kg, IV, on day 1), carboplatin (AUC 5 mg/mL/min, IV, on day 1), and etoposide (100 mg/m^2^, on days 1–3), every 3 weeks (Q3W), followed by QL1706 (5 mg/kg, Q3W) maintenance treatment until disease progression (PD), intolerable toxicity, death, consent withdrawal, completion of 24 months of treatment, or other discontinuation criteria defined in the study protocol (whichever occurred first). Dose modification for etoposide and carboplatin was allowed according to the guidelines described in the study protocol (Version 1.0, 13 February 2022). Dose modification for QL1706 was not permitted. QL1706 treatment continuation beyond PD was allowed at the investigator’s discretion, if sustained clinical benefit was observed.

### 2.4 Assessments

Safety was monitored from the signing of the informed consent form and throughout the treatment period until 90 days after the last dose of study treatment. Adverse events were graded according to National Cancer Institute Common Terminology Criteria for Adverse Events version 5.0 (CTCAE v5.0) and recorded. Tumor response was assessed by investigators using computed tomography or magnetic resonance imaging at baseline, every 6 weeks through week 48, and every 9 weeks thereafter. Tumor PD-L1 expression was analyzed using the VENTANA PD-L1 (SP263) assay (Roche) at the KingMed Diagnostics testing laboratory (Guangzhou, China). The combined positive score (CPS) of PD-L1 expression is calculated as the percentage of PD-L1 positive cells (tumor cells, lymphocytes, and macrophages) relative to the total number of viable tumor cells, multiplied by 100. Pre-treatment patient characteristics and laboratory data including complete blood cell count and LDH were collected. Pre-treatment LIPI was developed by combining a derived neutrophils/(leukocytes minus neutrophils) ratio (dNLR) greater than 3 and LDH greater than ULN (25). The LIPI score stratified patients to three groups: good LIPI (0 risk factors, dNLR ≤3 and LDH ≤ULN), intermediate LIPI (1 risk factor, dNLR >3 or LDH >ULN), and poor LIPI (2 risk factors, dNLR >3 and LDH >ULN) (26). Demographic, clinical, and pathological data were also collected.

### 2.5 Endpoints

The primary endpoint was safety. Secondary endpoints included the confirmed ORR, defined as the proportion of patients with complete response (CR) or partial response (PR) on two consecutive evaluations at least 4–6 weeks apart; disease control rate (DCR), defined as the proportion of patients with CR, PR, or stable disease (SD) lasting for at least one additional evaluation cycle as the best overall response; median progression-free survival (PFS), defined as the time from the first administration of study treatment to the first documented PD (radiologically confirmed per RECIST 1.1 criteria) or death, whichever occurred first; OS, defined as the time from initiation of study treatment to death; duration of response (DoR), defined as the time from the first documentation of a confirmed objective response (CR or PR) to PD or death from any cause, whichever occurred first.

### 2.6 Statistical analyses

This phase 2 exploratory study has safety as the primary endpoint. A total of 40 patients were planned to enable clinically meaningful safety assessment, not based on formal hypothesis testing. Assuming a true adverse event incidence of 5%, the probability of observing ≥1 event in 40 patients is ~87%, supporting detection of AEs with ≥5% incidence. The sample size also allows preliminary AE rate estimation with acceptable precision. No formal interim analysis was planned.

The full analysis set (FAS) were established in strict adherence to the intention-to-treat principle and included all patients who received at least 1 dose of study treatment and had a pathologically confirmed diagnosis of SCLC. Safety analyses were performed in the safety set (SS), which included all patients who received at least 1 dose of study treatment and underwent ≥1 post-treatment safety assessment. Efficacy analyses were primarily performed in the efficacy evaluable analysis set (EEAS), which included all patients in the FAS who had at least one post-baseline tumor assessment. *Post-hoc* exploratory analyses were performed to assess the benefit of QL1706 in patients who entered the maintenance phase. The QL1706 maintenance treatment was defined as administration of 4–6 cycles of QL1706 with etoposide-platinum followed by QL1706 monotherapy.

Continuous variables were described using median (95% CI) or mean (standard error, SE), and categorical variables using frequencies (percentages). ORR and DCR were descriptively summarized as percentages with 95% CIs calculated using the Clopper-Pearson method. Median time-to-event endpoints were estimated using the Kaplan-Meier method, and the corresponding 95% CI was derived using the Brookmeyer-Crowley method. The PFS and OS in patients stratified by LIPI were *post-hoc* compared using log-rank test. The best percentage change in target lesion size from baseline was presented with a waterfall plot. Duration of treatment and response in patients who had at least one evaluation of SD or better were summarized in a swimmer plot. Safety data were summarized descriptively. All statistical analyses were performed using the Statistical Analysis System version 9.4 (SAS Institute, Cary, NC).

## 3 Results

### 3.1 Patients

**Figure 1:**
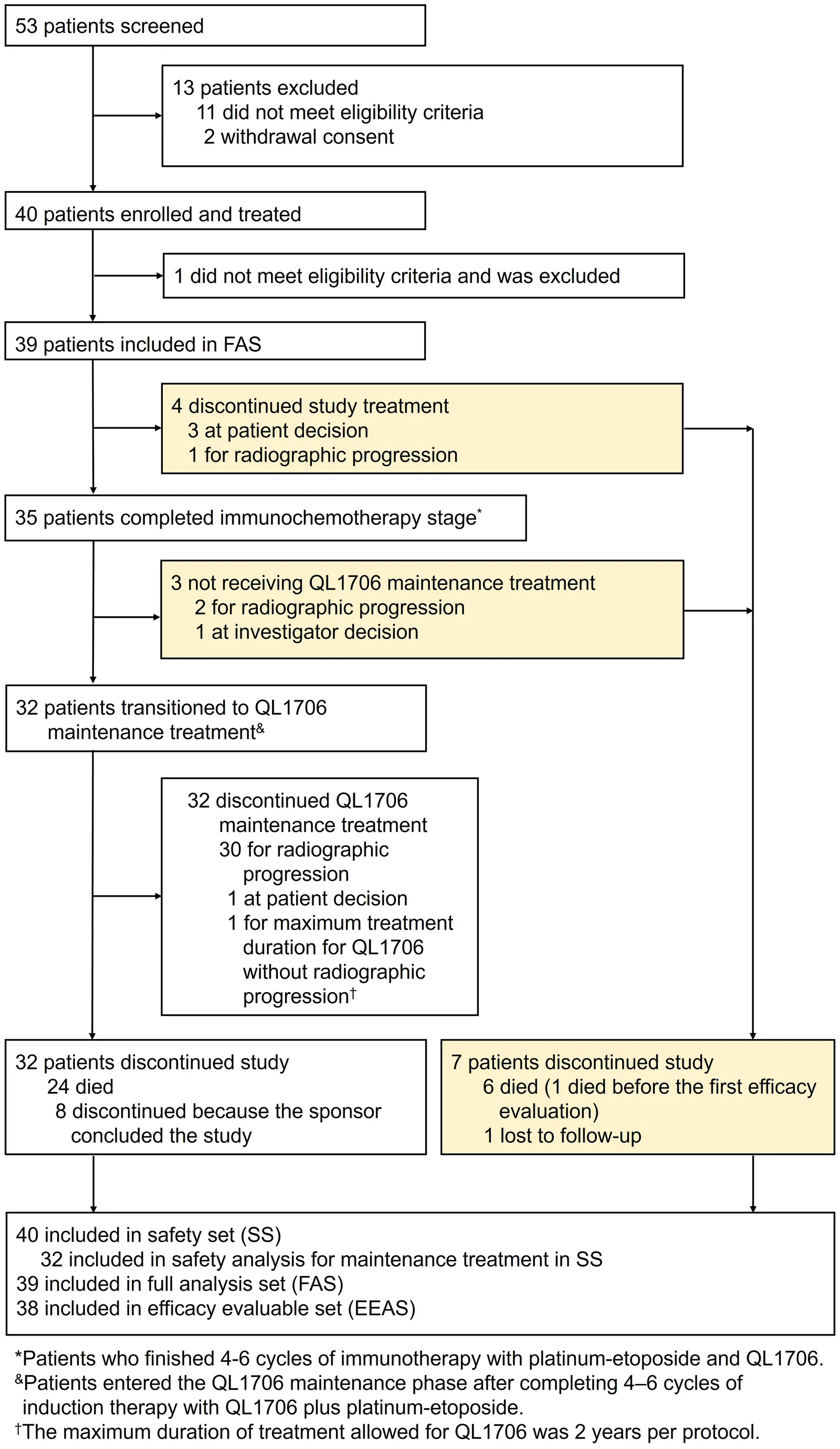
Trial profile. EEAS, efficacy evaluable analysis set; FAS, full analysis set; SS, safety set.

From 11 April 2022 to 25 July 2022, 53 patients were screened, of whom 13 patients were excluded and 40 patients were enrolled. Although all 40 patients had received at least one dose of study treatment, 39 patients were included in the FAS, as one patient was excluded due to an ineligible pathological diagnosis. In the FAS, a total of 35/39 patients (89.7%) had received 4–6 cycles of QL1706 combination treatment. Four patients (10.3%) discontinued the chemoimmunotherapy, one due to radiographic disease progression and three due to patient decision. A total of 32 patients (82.1%) received the scheduled subsequent QL1706 maintenance treatment. Three patients did not continue the subsequent QL1706 maintenance treatment, two due to radiographic disease progression and 1 due to investigator decision. At the time of this final analysis (16 December 2024), all 32 patients had discontinued study treatment due to radiographic disease progression (30/39 [76.9%]), due to patient’s decision for the COVID-19 pandemic (1/39 [2.6%]), or completion of 24-month treatment as prespecified in the protocol (1/39 [2.6%]). After a median follow-up duration of 26.6 months (interquartile range [IQR] 25.6–28.1), all patients discontinued the study. At the end of the study, 30/39 patients (76.9%) had died and 1/39 (2.6%) patient was lost to follow-up (**Figure 1**). Eight patients remained on study until the sponsor concluded the study.

The baseline characteristics are shown in **Table 1**. The median age was 58 years (range 38–73) and 87.2% of patients were male. Thirty-six patients (92.3%) had an ECOG performance status of 1 and 31 (79.5%) of patients were current or former smokers. All patients were diagnosed with ES-SCLC and 35 patients (89.7%) had stage IV disease (AJCC 8th edition). The majority of patients had PD-L1-negative tumors (CPS<1, 76.9%). Based on the baseline LIPI, 20 patients (51.3%) were classified as good, 17 (43.6%) as intermediate, and 2 (5.1%) as poor.

**Table 1:** Baseline demographics and disease characteristics.

Baseline characteristics	QL1706+Carboplatin+Etoposide (N = 39)
Age, median, years	58.0 (38–73)
<65 years, n (%)	26 (66.7)
≥65 years, n (%)	13 (33.3)
Sex, n (%)	
Male	34 (87.2)
Female	5 (12.8)
ECOG performance status, n (%)	
0	3 (7.7)
1	36 (92.3)
Smoking status, n (%)	
Current or former smoker	31 (79.5)
Never smoked	8 (20.5)
VALG stage at study entry, n (%)	
Extensive-stage	39 (100)
Clinical stage at study entry^1^, n (%)	
III	4 (10.3)
IV	35 (89.7)
PD-L1 expression (CPS), n (%)	
CPS<1	30 (76.9)
CPS≥1	9 (23.1)
Baseline lung immune prognostic index, n (%)	
Good	20 (51.3)
Intermediate	17 (43.6)
poor	2 (5.1)
Sum of longest diameter of target lesions, median, mm	105.3 (41.0–235.7)
Bone metastases, n (%)	12 (30.8)
Liver metastases, n (%)	9 (23.1)
Neutrophil-to-lymphocyte ratio^2^, median	2.7 (1.0–6.9)

CPS, combined positive score; ECOG, Eastern Cooperative Oncology Group; PD-L1, programmed death ligand 1; VALG, Veterans Administration Lung Study Group. Note: Staging was according to the eighth edition of the American Joint Committee on Cancer (AJCC) staging manual; Neutrophil-to-lymphocyte ratio=baseline neutrophil count/baseline lymphocyte count.

### 3.2 Treatment exposure

As of database lock on December 16, 2024, patients received a median of 8.0 treatment cycles(range 1–35) of QL1706 over a median treatment duration of 6.83 months (range0.7–24.8), and 85% of patients completed at least 6 cycles of QL1706 treatment. For the etoposide–carboplatin combination therapy, the median number of cycles administered was 6.0 (range 1–6) over 4.14 months (IQR 3.06–4.63) (**Supplementary Table 2**).

The conduct of our study was significantly affected by the COVID-19 pandemic, with 19 patients(47.5%) delaying QL1706 and 3 patients (7.5%) delaying chemotherapy due to COVID-19 infection orpandemic-related restrictions. The median treatment delay was 13.5 days (range 4–81) for QL1706 and 24 days (range 14–41) for chemotherapy (**Supplementary Table 3**). Although prophylactic cranial irradiation (PCI) to prevent intracranial metastases was allowed per protocol, no patient received PCI.

### 3.3 Safety

Forty patients were included in the SS. TRAEs of any grade occurred in 40 patients (100%). Grade 3–4 TRAEs occurred in 35 patients (87.5%) (**Table 2**). The most common grade 3–4 TRAEs were hematological toxicities, including neutrophil count decreased (72.5%), platelet count decreased (50%), and white blood cell (WBC) count decreased (47.5%). There were no treatment-related deaths.

**Table 2:** Summary of adverse events in safety analysis set.

Events, n (%)	QL1706+Carboplatin+Etoposide (N = 40), n (%)	
	Any grade	Grade 3–4
TRAEs	40 (100)	35 (87.5)
TRAEs leading to dose reduction of chemotherapy	11 (27.5)	11 (27.5)
TRAEs leading to any drug interruption	17 (42.5)	10 (25.0)
TRAEs leading to QL1706 interruption	13 (32.5)	7 (17.5)
TRAE leading to any drug discontinuation	0	0
Treatment-related serious adverse events	19 (47.5)	18 (45.0)
TRAEs in >10% in all patients		
Anemia	37 (92.5)	16 (40.0)
WBC count decreased	37 (92.5)	19 (47.5)
Neutrophil count decreased	35 (87.5)	29 (72.5)
Platelet count decreased	35 (87.5)	20 (50.0)
Nausea	26 (65.0)	0
Appetite decreased	15 (37.5)	0
Alopecia	15 (37.5)	0
Vomiting	15 (37.5)	1 (2.5)
ALT increased	15 (37.5)	1 (2.5)
Asthenia	15 (37.5)	1 (2.5)
AST increased	13 (32.5)	2 (5.0)
Constipation	11 (27.5)	0
Blood bilirubin increased	8 (20.0)	0
Hyponatremia	8 (20.0)	0
Proteinuria	8 (20.0)	0
Hypoalbuminemia	7 (17.5)	0
Hypertriglyceridemia	7 (17.5)	1 (2.5)
γ–glutamyl transferase increased	6 (15.0)	2 (5.0)
Body weight decreased	6 (15.0)	1 (2.5)
Blood creatinine increased	5 (12.5)	0
Pruritus	5 (12.5)	0
Rash	5 (12.5)	1 (2.5)
Hyperthyroidism	5 (12.5)	0
Hypothyroidism	5 (12.5)	0

ALT, alanine aminotransferase; AST, aspartate aminotransferase; TRAEs, treatment related adverse events; WBC, white blood cell. Note: TRAEs occurred in ≥10% patients are presented; TRAEs were those related to QL1706 or chemotherapy.

Treatment-related serious adverse events (TRSAEs) occurred in 19 patients (47.5%), most of whichwere hematologic toxicities caused by chemotherapy (**Supplementary Table 4**). TRAEs leading to QL1706 treatment interruption occurred in 13 patients (32.5%). Notably, no patients experienced TRAEs leading to treatment discontinuation during the study period. Immune-related AEs (irAEs) as assessed by investigators were observed in nine patients (22.5%), most of which were grade 1–2 (**Table 3**). Among the 9 patients who developed irAEs, the median time from QL1706 initiation to the first irAE was 3.1 months (range 0.1–11.0), and the median duration of irAEs was 1.3 months (range 0.1–10.7). The most frequent irAEs were rash (7.5%) and hypothyroidism (7.5%). Two patients (5.0%) experienced grade 3 irAEs, one with rash and one with vomiting. Notably, the grade 3 vomiting episode occurred during QL1706 maintenance therapy and was effectively controlled with standard antiemetic treatment, without corticosteroid intervention. The grade 3 irAE rash improved to grade 1 after treatment interruption and symptomatic treatment with loratadine tablets, topical compound dexamethasone acetate cream, and oral prednisone acetate tablets. One patient experienced grade 2 immune-mediated lung disease after 6 study treatment cycles. The diagnosis was supported by negative infectious workup, radiographic tumor shrinkage, and no prior thoracic radiotherapy, and the event improved to grade 1 after treatment interruption and glucocorticoid therapy. One patient (2.5%) experienced infusion-related reaction associated with QL1706, which was grade 1 dizziness event.

**Table 3:** Summary of immune-related adverse events and infusion-related reactions in safety analysis set.

Events, n (%)	QL1706+Carboplatin+Etoposide (N = 40), n (%)	
	Any grade	Grade 3
Immune-related AEs	9 (22.5)	2 (5.0)
Rash	3 (7.5)	1 (2.5)
Hypothyroidism	3 (7.5)	0
Blood TSH decreased	2 (5.0)	0
Pruritus	2 (5.0)	0
Blood ACTH decreased	1 (2.5)	0
Platelet count decreased	1 (2.5)	0
Blood bilirubin increased	1 (2.5)	0
Hyperthyroidism	1 (2.5)	0
Vomiting	1 (2.5)	1 (2.5)
Adrenal insufficiency	1 (2.5)	0
Generalized edema	1 (2.5)	0
Asthenia	1 (2.5)	0
Immune-mediated lung disease	1 (2.5)	0
Infusion-related reactions	2 (5.0)	0
Dizziness	1 (2.5)	0
Phlebitis	1 (2.5)	0

ACTH, adrenocorticotropic hormone; AEs, adverse events; TSH, thyroid stimulating hormone.

Most patients (32/40, 80%) in the SS entered the maintenance phase of QL1706 after completing4–6 cycles of combination therapy. The incidence of QL1706-related TRAEs during thecombination or maintenance phases has been summarized in **Supplementary Table 5**. During the maintenance phase, 19 patients (59.4%) experienced at least one QL1706-related TRAE, and 5 patients (15.6%) had grade 3–4 events. The incidence of QL1706-related TRAEs with a frequency of >10% was much lower in the maintenance phase than in the combination phase, particularly for grade 3–4 events.

### 3.4 Efficacy

**Figure 2:**
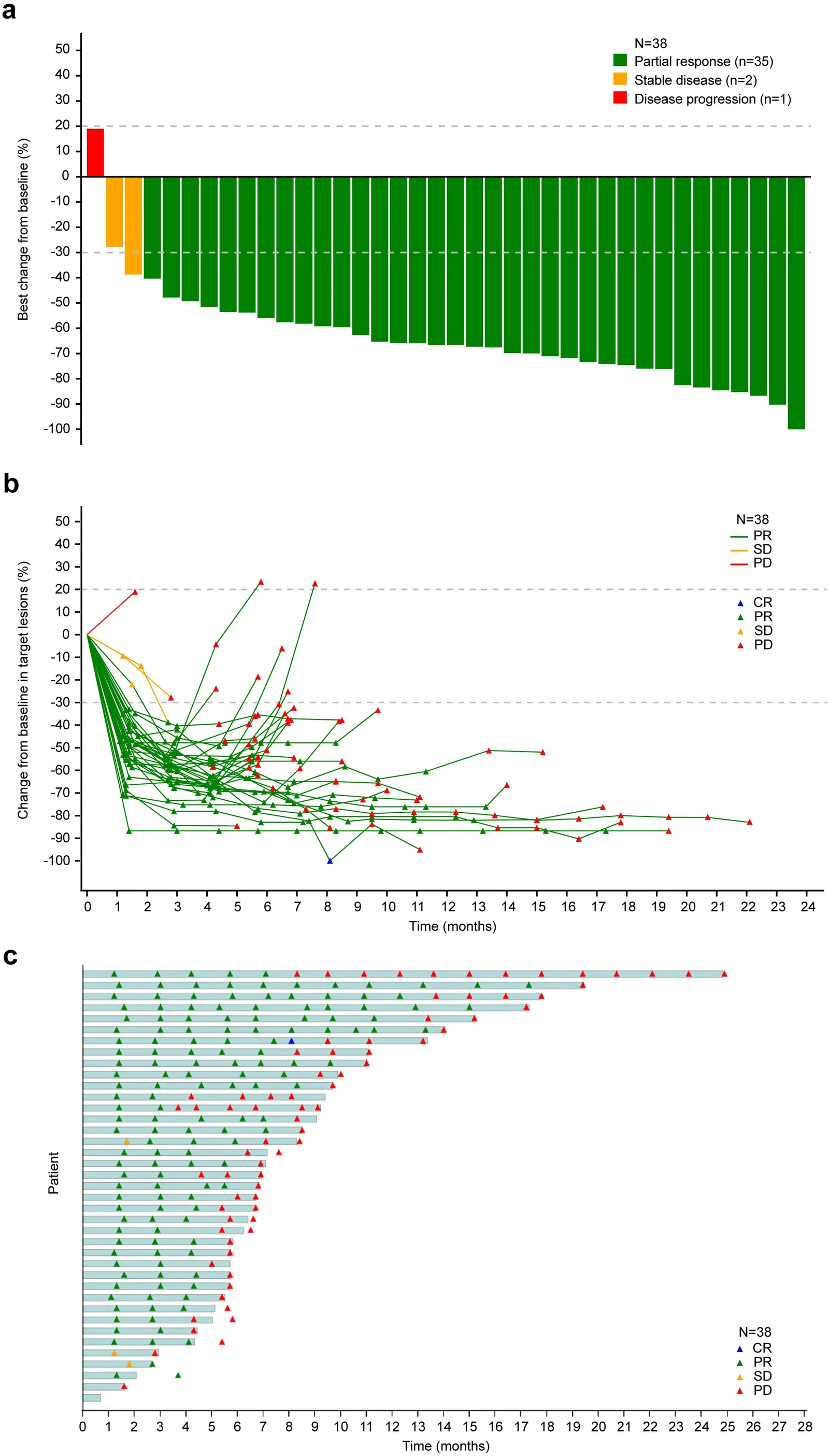
Tumor responses in EEAS. **(a)** Waterfall plots of the best percentage changes from baseline for the target lesions (RECIST version 1.1). **(b)** Spider plot of changes from baseline for the target lesions (RECIST version 1.1) over time. The dotted line in a represents the ORR in all patients in EEAS. **(c)** Swimmer plot of individual responses and treatment duration. CR, complete response; PD, progressive disease; PR, partial response; SD, stable disease; RECIST, Response Evaluation Criteria in Solid Tumors.

In the FAS, one patient was excluded from the EEAS due to the lack of treatment tumor evaluation. Consequently, efficacy analyses were performed on the remaining 38 patients. In the EEAS (n=38), 35 patients achieved confirmed PR, resulting in a confirmed ORR of 92.1% (95% CI 78.6–98.3) (**Figure 2a**; **Table 4**). The DCR was 97.4% (95% CI 86.2–99.9). SD was confirmed in two patients. Among them, one demonstrated PR at the final evaluation; however, since confirmation was precluded by COVID-19-related withdrawal, this patient was classified as SD according to RECIST v1.1. Tumor shrinkage of >30% in target lesions was observed in 37 patients (97.4%) (**Figure 2b**). The median best percentage change from baseline in target lesion was -66.7% (range -100% to +19.0%). The median DoR was 4.6 months (95% CI 4.2–6.9) (**Figure 2c**). Subgroup analyses of ORR are shown in **Supplementary Figure 1**. The ORRs in each subgroup were consistent with the overall ORR.

**Table 4:** Treatment response per response evaluation criteria in solid tumors v1.1 by investigator in efficacy evaluable analysis set.

Treatment response	QL1706+Carboplatin+Etoposide (N = 38)
Best overall response	
Confirmed partial response, n (%)	35 (92.1)
Stable disease, n (%)	2 (5.3)
Progressive disease, n (%)	1 (2.6)
Confirmed ORR, n (%)	35 (92.1)
95% CI	78.6–98.3
Disease control rate, n (%)	37 (97.4)
95% CI	86.2–99.9
Duration of response, months, median (95% CI)	4.6 (4.2–6.9)

The 95% CI was calculated based on Clopper-Pearson exact interval. CI, confidence interval; ORR, objective response rate.

**Figure 3:**
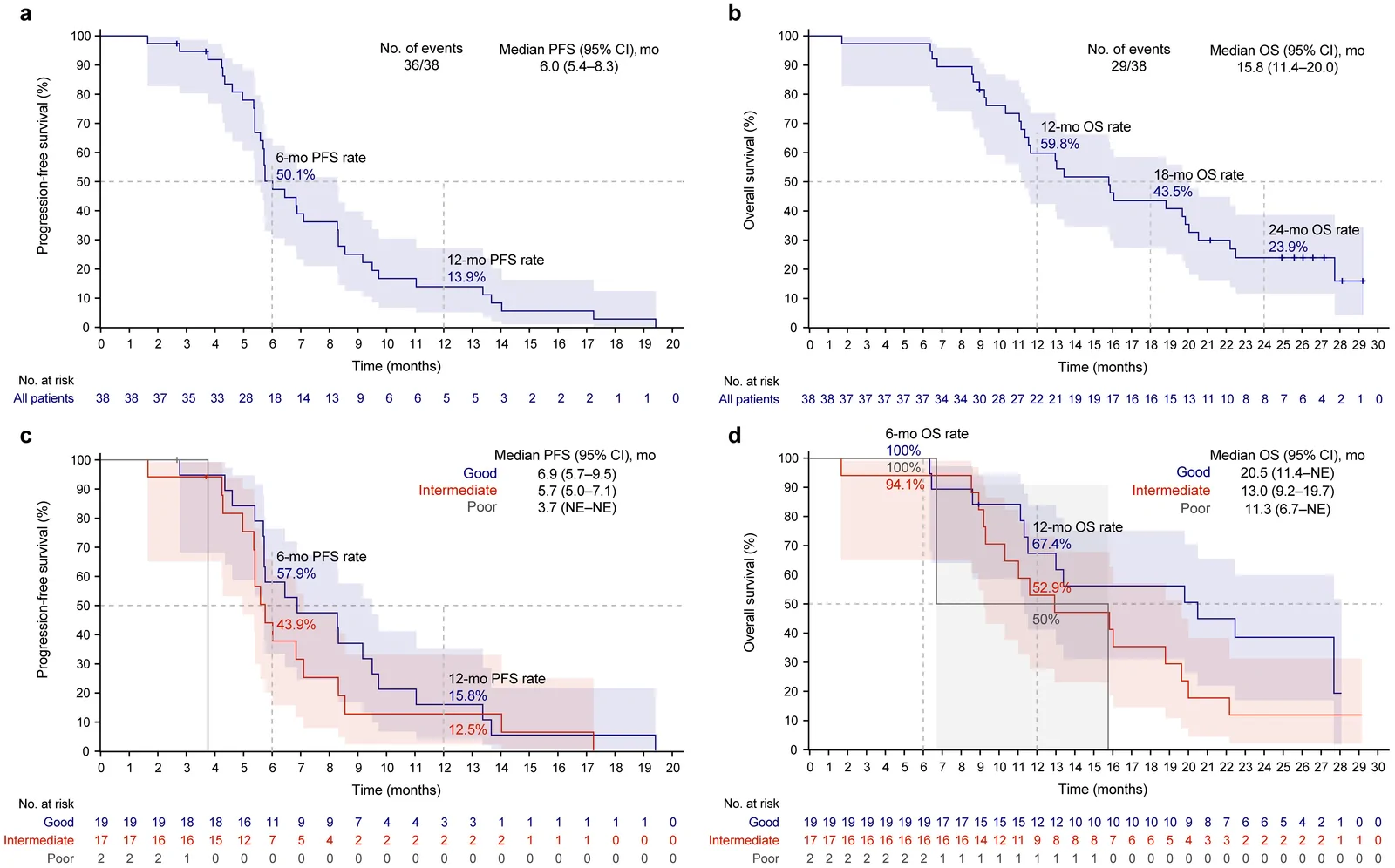
Kaplan-Meier curves of PFS and OS in EEAS and in patients stratified by LIPI. **(a, b)** Kaplan–Meier curves of PFS **(a)** and OS **(b)** in EEAS; **(c, d)** Kaplan–Meier curves of PFS **(c)** and OS **(d)** in patients stratified by baseline LIPI in EEAS. EEAS, efficacy-evaluable analysis set; PFS, progression-free survival; LIPI, lung immune prognostic index; OS, overall survival.

The median PFS was 6.0 months (95% CI 5.4–8.3), with a 6-month PFS rate of 50.1% (**Figure 3a**). In the EEAS, 36 patients were assessed as having PD, while two patients were censored dueto missing two scheduled efficacy assessments. The tumor progression pattern in the 36 patients aresummarized in **Supplementary Table 6**. Brain metastases were the most common new lesions (33.3%, 4 of 12 patients with new lesions). Tumor shrinkage of >30% in target lesions from baseline was sustained throughout the treatment period in 29 patients (76.3%), even beyond radiographic disease progression (**Figure 3b**).

At the data cutoff date, 29 patients (76.3%) had died. The median OS was 15.8 months (95% CI 11.4–20.0) (**Figure 3b**). The OS rates were 59.8% (95% CI 42.4–73.5), 43.5% (95% CI 27.4–58.6), and 23.9% (95% CI 11.6–38.6) at 12, 18, and 24 months, respectively. When stratified by baseline LIPI status, patients with good LIPI (n=19) had a favorable median PFS of 6.9 months (95% CI 5.7–9.5) and median OS of 20.5 months (95% CI 11.4–not evaluable) (**Figures 3c, d**). In the OS subgroup analyses stratified by PD-L1 expression, the median OS was 19.7 months(95% CI 11.4–22.5) in the PD-L1 CPS <1 group (n=30) and 11.6 months (95% CI6.7–13.4) in the PD-L1 CPS ≥1 group (n=9) (**Supplementary Figure 2**). Among the eight patients who received ≥2 cycles of study treatment beyond diseaseprogression, the median OS was 23.8 months (95% CI 11.2–not evaluable) (**Supplementary Figure 3**).

In the FAS, 28 patients (71.8%) received at least one subsequent anticancer therapy (**Supplementary Table 7**). The most common treatments included chemotherapy (53.8%), targeted therapy (25.6%), and immunotherapy (20.5%) (including 5 patients who received other PD-1/PD-L1 inhibitors).

## 4 Discussion

In this open-label, single-arm, phase 2 study, QL1706 showed a good safety profile with no unexpected safety signals and no TRAEs leading to treatment discontinuation in both combination and maintenance treatment stages. QL1706 and chemotherapy yielded a confirmed ORR of 92.1%, a median PFS of 6.0 months and a median OS of 15.8 months in first-line treatment of ES-SCLC. In *post-hoc* exploratory analyses, ES-SCLC patients with a good LIPI status showed better clinical benefit from QL1706 plus chemotherapy than patients with intermediate or poor LIPI status; the median OS in ES-SCLC patients with PD-L1 CPS <1 was numerically longer than that in patients with PD-L1 CPS ≥1.

In this study, QL1706 combined with 4–6 cycles of chemotherapy showed a favorable safety profile. The exposure levels of QL1706 and chemotherapy agents were relatively high. Patients received relatively high cumulative doses of both, with a median of eight QL1706 doses, higher than that reported for durvalumab (6 [IQR 4–10]) and tremelimumab (5 [IQR 4–5]) in the durvalumab plus tremelimumab plus chemotherapy arm in the CASPIAN study (12, 14). The median duration of chemotherapy was also longer (16.91 weeks vs 12.3 weeks), which may have contributed to the observed efficacy without compromising tolerability. The most frequently observed AEs were hematologic toxicities, including anemia, decreased WBC count, decreased neutrophil count, and decreased platelet count. These events predominantly occurred during the combination stage and were mainly attributed to chemotherapy. Notably, severe toxicities were less frequent during QL1706 maintenance therapy. Among the 32 patients who received QL1706 maintenance treatment, grade 3–4 TRAEs were reported in only five patients (15.6%), reinforcing the favorable tolerability of QL1706 in the maintenance setting. The incidence of grade 3–4 TRAEs during the maintenance treatment was similar to that associated with long-term QL1706 monotherapy in the overall solid tumor population in the phase I study (23, 25, 27). In terms of irAEs, the current study showed a lower incidence rate of grade 3–4 irAEs (5% *vs* 14%), a lower treatment discontinuation rate (0% *vs* 16%), and a lower treatment-related death rate (0% *vs* 5%), compared with the durvalumab plus tremelimumab plus chemotherapy arm in the CASPIAN study (14). These findings indicate a favorable tolerability profile of QL1706 plus chemotherapy in first-line treatment of ES-SCLC. However, cross-trial comparison should be interpreted cautiously.

The baseline characteristics of the current study population were generally comparable to those reported in previous studies, with a relatively higher proportion of patients with ECOG performance status 1 (92.3% vs 59%-86%) (5–9). The current study was conducted in China and enrolled patients, of whom 20.5% were never smokers. This proportion was similar to the 20%–23% reported among Asian patients, but higher than the 5%–6% observed in non-Asian populations in previous phase 3 studies of first-line treatment for ES-SCLC (5–9). Since SCLC is a smoking-related malignancy, a history of smoking was associated with poorer treatment outcomes (28, 29). Notwithstanding the challenge, the QL1706 plus chemotherapy regimen exhibited notable anti-tumor activity, with an ORR of 92.1% in the first-line treatment of ES-SCLC. The efficacy of chemoimmunotherapy regimens incorporating PD-1/PD-L1 inhibitors has been reported to achieve an ORR of 60.2%–80.2% in published trials (4, 6–9, 11).

The encouraging tumor response with QL1706 combination therapy enabled most patients to receive QL1706 maintenance treatment, thereby improving the likelihood of continuing QL1706 therapy beyond PD. The promising efficacy may be attributed to two key factors. First, the favorable tolerability allowed for longer duration of QL1706 combination and maintenance treatments. Notably, 85% of patients completed at least 6 cycles of QL1706 treatment (median, 8 cycles), whereas only 61% of patients completed the 5 planned doses of tremelimumab in the CASPIAN study. Second, continuing therapy beyond PD may provide additional clinical benefit, as supported by the extended median OS of 23.8 months in patients who received at least 2 treatment cycles following confirmed PD. Compared with the survival outcomes reported in the IMpower133 trial, the QL1706-based regimen in this study achieved a more favorable median PFS (6.0 months vs 5.2 months) and median OS (15.8 months vs 12.3 months) (6). However, the OS benefit should be interpreted cautiously, as the median OS may be affected by the subsequent anti-tumor therapy. The encouraging preliminary results warrant large-scale, randomized, controlled studies to validate the efficacy and safety of QL1706 plus chemotherapy as first-line treatment for ES-SCLC.

In the *post-hoc* exploratory subgroup analysis stratified by LIPI status, patients with a good LIPI status appeared to derive greater clinical benefit from QL1706-based chemoimmunotherapy compared with those with intermediate or poor LIPI status. LIPI, an easily accessible clinical index based on baseline dNLR and LDH, has been proposed as a prognostic biomarker of immunotherapy in advanced NSCLC and metastatic renal cell carcinoma (26, 30). The prognostic relevance of LIPI in ES-SCLC has also been indicated in a retrospective study in patients who received atezolizumab plus chemotherapy as first-line treatment (22). The findings of the present study are consistent with these previous observations (22, 26, 30).

This study indicated that PD-L1 negativity was associated with enhanced survival benefit from QL1706-based dual PD-1/CTLA-4 immunotherapy. The observation that patients with PD-L1-negative ES-SCLC may derive benefit from dual PD-1 and CTLA-4 inhibition with or without chemotherapy is consistent with findings reported in non-small cell lung cancer (NSCLC) and melanoma (31–33). In a pooled analysis of CheckMate-9LA and CheckMate227, dual PD-1/CTLA-4 inhibition with or without chemotherapy was associated with a substantial and durable overall survival benefit versus chemotherapy in patients with metastatic NSCLC and PD-L1 expression <1% (HR 0.64) (31). In CheckMate-067, nivolumab plus ipilimumab has also demonstrated durable long-term benefit over nivolumab monotherapy in the PD-L1-negative subgroup of patients with advanced melanoma (32, 33). Together, dual immunotherapy targeting PD-1 and CTLA-4 may exhibit promising efficacy in PD-L1-negative patients across tumor types.

The promising efficacy of QL1706 combination therapy, including the numerically longer OS in the PD-L1-negative subgroup, may be related to its unique pharmacological mechanisms. QL1706 contains the anti-PD-1 IgG4 antibody iparomlimab, which targets PD-1 and blocks immunosuppressive signals mediated by both PD-L1 and PD-L2. In contrast, durvalumab targets only PD-L1 and does not inhibit PD-L2-mediated immune evasion, which may limit CTLA-4-mediated T-cell activation (34). This difference may partly account for the favorable efficacy observed with QL1706 compared with the durvalumab plus tremelimumab and EP arm in the CASPIAN trial. Mechanistically, anti-PD-L1 antibodies can disrupt the cis-PD-L1–CD80 complex on antigen-presenting cells, leading to CD80 homodimer formation, CTLA-4-mediated depletion, and attenuated CD28 co-stimulation, whereas anti-PD-1 antibodies preserve this cis-interaction and maintain CD28 co-stimulation (35). Consequently, in PD-L1-negative tumors, QL1706 may preserve CD80 levels and enhance CD28-mediated T-cell co-stimulation. PD-L1-negative but PD-L2-positive tumors may still benefit from anti-PD-1 therapy through concurrent PD-L2/PD-1 axis blockade. Furthermore, PD-L1 expression alone may not fully reflect an active tumor immune microenvironment, as immunotherapy responses can also be influenced by higher TMB (36) or enhanced neoantigen presentation, promoting adaptive anti-tumor immunity (37).

However, there were some limitations in our study. First, the lack of a head-to-head comparison with the current standard of care precludes definitive conclusions regarding the relative efficacy of the QL1706 regimen. Second, the promising OS outcome should be interpreted cautiously, as it might have been confounded by subsequent anti-tumor therapy. Third, the small sample size may have underpowered the analyses, so the results should be interpreted cautiously, particularly in subgroups with potential imbalance. Fourth, from a methodological standpoint, despite being protocol-permitted, the omission of PCI may have contributed to the observed predominance of brain metastases as a new lesion site. Finally, the COVID-19 pandemic significantly disrupted treatment schedules, leading to delays or discontinuations that may have affected the observed efficacy of the QL1706 chemoimmunotherapy.

In conclusion, QL1706 plus EC was well tolerated with no new safety signals and showed promising anti-tumor activity as a first-line treatment for ES-SCLC, suggesting that it may represent a potential therapeutic option for this patient population.

## Data Availability

The raw data supporting the conclusions of this article will be made available by the authors, without undue reservation.
