# Supplementary material for: Safety and efficacy of first-line iparomlimab and tuvonralimab (QL1706) plus carboplatin and etoposide in patients with extensive-stage small cell lung cancer: an open-label, single-arm, phase 2 trial (DUBHE-L-209)
Source: Front Immunol. 2026 Aug 11;17:1879524. doi: 10.3389/fimmu.2026.1879524 (PMC13503353; doi:10.3389/fimmu.2026.1879524)
Supplement: Supplementary file 1 [file DataSheet1.docx]

Supplementary Material

First-line iparomlimab and tuvonralimab (QL1706) plus carboplatin and etoposide in patients with extensive-stage small cell lung cancer: an open-label, single-arm, phase 2 trial (DUBHE-L-209)

**This document includes:**

Supplementary Tables: Table S1 to S7

Supplementary Figures: Figure S1 to S3

# 1 Supplementary Figures and Tables

## 1.1 Supplementary Figures

**Figure S1.** Subgroup analyses of objective response rate in efficacy evaluable analysis set. Abbreviations: ECOG PS, Eastern Cooperative Oncology Group Performance status; CPS, combined positive score; PD-L1, programmed death ligand 1; TPS, tumor proportion score.

**Figure S2.** Kaplan‒Meier curve of overall survival in patients categorized by baseline CPS. Abbreviation: CPS, combined positive score.

**Figure 3.** Kaplan‒Meier curve of overall survival in patients who received ≥2 cycles vs. <2 cycle of study treatment beyond disease progression.

## 1.2 Supplementary Tables

**Table S1.** Trial Inclusion/Exclusion Criteria‌

**1. Inclusion Criteria**‌

Patients must meet ‌all‌ of the following criteria to be eligible:

1. Aged 18–75 years, male or female.
2. Histologically or cytologically confirmed extensive-stage small-cell lung cancer (ES-SCLC) (staged per Veterans Administration Lung Study Group [VALG] criteria).
3. ECOG performance status 0–1.
4. No prior first-line systemic therapy or immune checkpoint inhibitors for ES-SCLC. Patients who underwent surgery with curative-intent adjuvant therapy (e.g., radiotherapy, chemotherapy) are eligible if ≥6 months have elapsed between the last adjuvant treatment and ES-SCLC diagnosis.
5. Must provide pre-treatment tumor tissue specimens (archived within 6 months prior to the first dose or freshly obtained). Specimens must be formalin-fixed, paraffin-embedded (FFPE) blocks yielding ≥5 slides (4–5 μm thickness) for biomarker testing. Exclusions: Fine-needle aspirates, pleural fluid cell pellets, decalcified bone specimens without soft tissue, or core biopsies with insufficient material. Note: Survival expectancy ≥12 weeks.
6. ≥1 measurable lesion per RECIST v1.1. Previously irradiated lesions must demonstrate post-radiation progression and cannot be the sole target lesion.
7. Women of childbearing potential (WOCBP) must have a negative serum pregnancy test within 7 days before enrollment. WOCBP and male patients with WOCBP partners must use contraception from signing informed consent until 24 weeks after the last dose (methods per protocol Section 4.3.1).
8. Laboratory values within 14 days before the first dose:

(1) Hematology (no transfusion/growth factors within 14 days): WBC ≥3.0 × 10^9/L, ANC ≥1.5 × 10^9/L, Platelets ≥100 × 10^9/L, Hemoglobin ≥9.0 g/dL.

(2) Liver function: AST/ALT ≤2.5 × ULN (without liver metastases); ≤5 × ULN (with liver metastases); Total bilirubin ≤1.5 × ULN (≤3.0 mg/dL if Gilbert’s syndrome).

(3) Renal function: Serum creatinine ≤1.5 × ULN or CrCl ≥50 mL/min (Cockcroft/Gault formula).

(4) Coagulation (if not on anticoagulants): INR/APTT ≤1.5 × ULN. Patients on stable anticoagulant doses are eligible.

(5) Other: Lipase/amylase ≤1.5 × ULN (allowed if >1.5 × ULN without clinical/radiographic pancreatitis), ALP ≤2.5 × ULN (≤5 × ULN with liver/bone metastases).

9. Voluntarily signs informed consent, demonstrates compliance, and agrees to follow-up.

**2. Exclusion Criteria**‌

Patients meeting ‌any‌ of the following are ineligible:

1. Active or untreated CNS metastases per screening/prior imaging (CT/MRI). Exceptions: Treated asymptomatic CNS metastases if: supratentorial/cerebellar only; no ongoing corticosteroids; stable post-CNS therapy; new asymptomatic lesions must receive radiation/surgery before enrollment.
2. Unresolved spinal cord compression or compression requiring steroids within 1 week pre-study.
3. Symptomatic third-space effusions requiring frequent drainage (e.g., pericardial/pleural/ascites uncontrolled by intervention).
4. Other active malignancies within ≤5 years, except: cured cervical carcinoma in situ, basal/squamous skin cancer, localized prostate cancer post-resection, or ductal carcinoma in situ. Hormonal therapy for non-metastatic prostate/breast cancer is permitted.
5. Active/suspected autoimmune diseases (e.g., myasthenia gravis, autoimmune hepatitis, SLE, RA, IBD). Exceptions: Type I diabetes (insulin-controlled), hypothyroidism on hormone replacement, vitiligo/psoriasis/eczema covering <10% body surface area (low-potency topical steroids only; no acute exacerbation within 12 months).
6. Prior T-cell co-stimulation/immune checkpoint therapy (e.g., anti-CTLA-4, anti-PD-1/PD-L1/PD-L2).
7. Systemic corticosteroids (>10 mg/day prednisone equivalent) or immunosuppressants within ≤14 days pre-dose. Allowed: Inhaled/topical steroids, physiologic replacement doses, or short-course steroids for prophylaxis (e.g., nausea).
8. Active infections: HBV DNA >1,000 copies/mL or 200 IU/mL (if HBsAg+); HCV RNA+ or HCV Ab+; Known HIV/AIDS.
9. Interstitial lung disease (e.g., idiopathic pulmonary fibrosis, organizing pneumonia) or active pneumonia requiring steroids; history of radiation pneumonitis is allowed.
10. Active tuberculosis (TB) or history of active TB within ≤48 weeks pre-screening.
11. Active systemic infection requiring IV antivirals/antibiotics/antifungals within 7 days pre-dose. Treatment must complete ≥1 week before enrollment.
12. Major surgery within 28 days pre-screening or planned during the study.
13. Live vaccines within 28 days pre-screening or expected during the study. Inactivated COVID-19 vaccines are permitted.
14. Significant cardiovascular disease: NYHA class ≥II heart failure, unstable angina/arrhythmia, uncontrolled hypertension, myocardial infarction, or stroke within ≤6 months.
15. Prior allogeneic bone marrow or solid organ transplantation.
16. Hypersensitivity to the study drug/excipients, severe allergy to monoclonal antibodies, or history of carboplatin/etoposide allergy.
17. Participation in another interventional trial or use of investigational drugs within 4 weeks pre-ICF signing.
18. Anticancer herbal medicines or immunomodulators (e.g., thymosin, interferon, interleukin) within 2 weeks pre-dose.
19. History of psychiatric disorders, alcohol/drug abuse, or inability to cease smoking.
20. Other factors deemed by investigators to compromise safety or protocol compliance (e.g., severe comorbidities, inadequate follow-up).

**Table S2.** Treatment exposure in full analysis set

| Treatment exposure | QL1706  (n=39) | Carboplatin  (n=39) | Etoposide  (n=39) |
| --- | --- | --- | --- |
| Number of treatment cycles |  |  |  |
| Median (range) | 8 (1–35) | 6 (1–6) | 6 (1–6) |
| Duration of treatment, months, |  |  |  |
| Median (range) | 6.83 (0.7–24.8) | 4.14 (0.7–5.7) | 4.14 (0.7–5.7) |
| Patients receiving ≥6 cycles of QL1706, n (%) | 33 (84.6) | NA | NA |
| Patients receiving ≥13 cycles of QL1706, n (%) | 10 (25.6) | NA | NA |
| Patients receiving 6 cycles of chemotherapy, n (%) | NA | 20 (51.3) | 20 (51.3) |
| Relative dose intensity, %, mean (SD) | 85.67 (11.943) | 82.93 (14.528) | 86.97 (14.742) |

Abbreviation: SD, standard deviation.

**Table S3. Treatment delay due to COVID-19 in safety analysis set**

| Treatment delay | QL1706  (n=40) | Carboplatin  (n=40) | Etoposide  (n=40) |
| --- | --- | --- | --- |
| Treatment delay | | | |
| Number of patients (%) | 19 (47.5) | 3 (7.5) | 3 (7.5) |
| Treatment delay time, days | | | |
| Mean (SD) | 20.0 (18.21) | 26.3 (13.65) | 26.3 (13.65) |
| Median (range) | 13.5 (4–81) | 24 (14–41) | 24 (14–41) |

Abbreviation: SD, standard deviation.

**Table S4. Summary of treatment-related serious adverse events in safety analysis set**

| SAE | QL1706-Related  (n=40) | Carboplatin-Related  (n=40) | Etoposide-Related  (n=40) | Total  (n=40) |
| --- | --- | --- | --- | --- |
| Any SAE | 8 (20.0) | 17 (42.5) | 17 (42.5) | 19 (47.5) |
| Platelet count decreased | 0 (0.0) | 9 (22.5) | 9 (22.5) | 9 (22.5) |
| Neutrophil count decreased | 0 (0.0) | 7 (17.5) | 7 (17.5) | 7 (17.5) |
| Anemia | 2 (5.0) | 5 (12.5) | 5 (12.5) | 5 (12.5) |
| WBC count decreased | 0 (0.0) | 2 (5.0) | 2 (5.0) | 2 (5.0) |
| AST increased | 1 (2.5) | 2 (5.0) | 2 (5.0) | 2 (5.0) |
| Febrile neutropenia | 0 (0.0) | 2 (5.0) | 2 (5.0) | 2 (5.0) |
| Pyrexia | 1 (2.5) | 1 (2.5) | 1 (2.5) | 1 (2.5) |
| Asthenia | 1 (2.5) | 0 (0.0) | 0 (0.0) | 1 (2.5) |
| Decreased body weight | 1 (2.5) | 1 (2.5) | 1 (2.5) | 1 (2.5) |
| ALT increased | 0 (0.0) | 1 (2.5) | 1 (2.5) | 1 (2.5) |
| Myocardial necrosis marker increased | 0 (0.0) | 1 (2.5) | 1 (2.5) | 1 (2.5) |
| Vomiting | 1 (2.5) | 0 (0.0) | 0 (0.0) | 1 (2.5) |
| Diarrhea | 1 (2.5) | 1 (2.5) | 1 (2.5) | 1 (2.5) |
| Diabetes | 1 (2.5) | 0 (0.0) | 0 (0.0) | 1 (2.5) |
| Adrenal insufficiency | 1 (2.5) | 0 (0.0) | 0 (0.0) | 1 (2.5) |
| Pneumonia | 0 (0.0) | 1 (2.5) | 1 (2.5) | 1 (2.5) |

Abbreviations: ALT, alanine aminotransferase; AST, aspartate aminotransferase; SAE, serious adverse event; WBC, white blood cell.

**Table S5. Summary of QL1706-related TRAEs in combination or maintenance phases in safety analysis set**

| Events, n (%) | QL1706+Carboplatin+Etoposide, n (%) | | | |
| --- | --- | --- | --- | --- |
|  | Combination Treatment (N=40) | | QL1706 Maintenance Treatment (N=32) | |
|  | Any grade | Grade 3-4 | Any grade | Grade 3-4 |
| QL1706-related TRAEs | 31 (77.5) | 16 (40.0) | 19 (59.4) | 5 (15.6) |
| TRAEs leading to QL1706 interruption | 10 (25.0) | 5 (12.5) | 5 (15.6) | 3 (9.4) |
| TRAEs leading to any drug discontinuation | 0 | 0 | 0 | 0 |
| Treatment-related serious adverse events | 6 (15.0) | 5 (12.5) | 3 (9.4) | 2 (6.3) |
| QL1706-related TRAEs occurring in >10% of patients during either the combination or maintenance phase | | | | |
| Anemia | 20 (50.0) | 5 (12.5) | 6 (18.8) | 0 |
| Platelet count decreased | 16 (40.0) | 3 (7.5) | 4 (12.5) | 0 |
| WBC count decreased | 12 (30.0) | 3 (7.5) | 6 (18.8) | 0 |
| Nausea | 12 (30.0) | 0 | 1 (3.1) | 0 |
| Neutrophil count decreased | 11 (27.5) | 8 (20.0) | 4 (12.5) | 1 (3.1) |
| Asthenia | 11 (27.5) | 0 | 1 (3.1) | 1 (3.1) |
| Alopecia | 10 (25.0) | 0 | 0 | 0 |
| Appetite decreased | 10 (25.0) | 0 | 1 (3.1) | 0 |
| ALT increased | 8 (20.0) | 1 (2.5) | 3 (9.4) | 0 |
| AST increased | 7 (17.5) | 2 (5.0) | 3 (9.4) | 0 |
| Proteinuria | 7 (17.5) | 0 | 3 (9.4) | 0 |
| Hypertriglyceridemia | 7 (17.5) | 0 | 3 (9.4) | 1 (3.1) |
| Blood bilirubin increased | 6 (15.0) | 0 | 2 (6.3) | 0 |
| Hypoalbuminemia | 5 (12.5) | 0 | 1 (3.1) | 0 |
| γ–glutamyl transferase increased | 5 (12.5) | 1 (2.5) | 0 | 0 |
| Vomiting | 5 (12.5) | 0 | 1 (3.1) | 1 (3.1) |
| Amylase increased | 5 (12.5) | 0 | 2 (6.3) | 0 |

Abbreviations: ALT, alanine aminotransferase; AST, aspartate aminotransferase; TRAEs, treatment-related adverse events; WBC, white blood cell.

**Table S6. Disease progression pattern in patients with disease progression according to Response Evaluation Criteria in Solid Tumors v1.1 in** **efficacy evaluable analysis set**

| **Disease progression** | **Total Patients in EEAS (N=38), n (%)** |
| --- | --- |
| Disease progression pattern |  |
| Target lesion only | 14 (36.8) |
| New lesion only | 12 (31.6) |
| Target lesion + non-target lesion + new lesion | 4 (10.5) |
| Target lesion + new lesion | 3 (7.9) |
| Non-target lesion + new lesion | 2 (5.3) |
| Non-target lesion only | 1 (2.6) |
| New lesion site, n (%) |  |
| Brain | 9 (23.7) |
| Lung | 4 (10.5) |
| Bone | 4 (10.5) |
| Lymph node | 3 (7.9) |
| Adrenal gland | 3 (7.9) |
| Liver | 2 (5.3) |

Abbreviations: EEAS, efficacy evaluable analysis set.

**Table S7. Subsequent anticancer therapy in full analysis set**

| **Subsequent anticancer therapy** | **QL1706+Carboplatin+Etoposide**  **(N=39), n (%)** |
| --- | --- |
| At least one subsequent anticancer therapy | 28 (71.8) |
| Chemotherapy | 21 (53.8) |
| Irinotecan | 7 (17.9) |
| Cisplatin | 6 (15.4) |
| Temozolomide | 5 (12.8) |
| Etoposide | 4 (10.3) |
| Paclitaxel | 4 (10.3) |
| Other anticancer drugs | 3 (7.7) |
| Nedaplatin | 3 (7.7) |
| Carboplatin | 1 (2.6) |
| Semustine | 1 (2.6) |
| Targeted therapy | 10 (25.6) |
| Anlotinib | 6 (15.4) |
| HTMC 0435 | 4 (10.3) |
| Immunotherapy | 8 (20.5) |
| RRX 001 | 2 (5.1) |
| IMM2520 | 1 (2.6) |
| PM 8002 | 1 (2.6) |
| TQB 2618 | 1 (2.6) |
| Sintilimab | 1 (2.6) |
| Envafolimab | 1 (2.6) |
| Serplulimab | 1 (2.6) |
| Tifcemalimab | 1 (2.6) |
| Penpulimab | 1 (2.6) |
| Toripalimab | 1 (2.6) |
| Traditional Chinese medicine | 3 (7.7) |
| B7H3 ADC | 1 (2.6) |
| Radiotherapy | 10 (25.6) |

Abbreviation: ADC, antibody-drug conjugate
